# Epidemiology of bovine fascioliasis in the Nile Delta region of Egypt: Its prevalence, evaluation of risk factors, and its economic significance

**DOI:** 10.14202/vetworld.2017.1241-1249

**Published:** 2017-10-17

**Authors:** Abdelgawad S. El-Tahawy, Eman K. Bazh, Reda E. Khalafalla

**Affiliations:** 1 Department of Animal Husbandry and Wealth Development, Faculty of Veterinary Medicine, Damanhour University, Egypt; 2 Department of Pathology and Parasitology, Faculty of Veterinary Medicine, Damanhour University, Egypt; 3 Department of Parasitology, Faculty of Veterinary Medicine, Kafrelsheikh University, Kafr El-Sheikh, Egypt

**Keywords:** cattle, Egypt, fascioliasis, Nile Delta, prevalence, risk factors

## Abstract

**Aim:**

This study focuses on the risk factors associated with the prevalence of *Fasciola* affecting cattle population in three provinces belonging to the Nile Delta of Egypt and to estimate the economic losses as a result of fascioliasis.

**Materials and Methods:**

From January 2015 to end of December 2015, records of 21 farms (4976 cattle) were analyzed to screen the prevalence of fascioliasis among cattle farms, to identify its associated risk factors and its economic impacts on Nile Delta region of Egypt.

**Results:**

The overall prevalence of fascioliasis in the Nile Delta region of Egypt was 9.77%. The prevalence of fascioliasis was found to be statistically significantly associated with age, sex, breed, and type of farms. The highest prevalence was observed in <2 age group (10.91%), and the lowest was >3 age groups (8.35%). In terms of body condition scores, cattle with medium and poor conditions were associated with fascioliasis more than those with good body condition. Besides, cattle raised in organic farms were associated with lower risk of fascioliasis than those in conventional farms. The prevalence of fascioliasis was noted more prominent in districts with moderate temperatures and with relative humidity (>60%). The annual overall costs for fascioliasis were estimated to be 221.2 USD/cow due to the significant reduction in body weight, reduction in milk production, and the treatment costs for fascioliasis.

**Conclusion:**

The results provided could be helpful for improving the control and preventive strategies.

## Introduction

In Egypt, the prevalence of parasitic diseases among farm animals varied according to many factors including irrigation, season and frequency of exposure of animal to infection, immune condition of the animal, the geographic location, and climatic conditions [1,2]. Parasitic infections among farm animals greatly affect livestock production and cause important economic losses including the retardation of growth, emaciation, and significant decrease in efficiency as well as the low production of milk, meat, and wool.

Certainly, the most pathogenic and economically important helminths are the liver flukes or fascioliasis where they cause traumatic hepatitis, peritonitis, and sudden death in acute fascioliasis. Fascioliasis is an important parasitic disease caused by *Fasciola* species (*Fasciola gigantica* and *Fasciola hepatica*) and found mostly in moderate weather areas worldwide with great influence on the world’s economy, due to its great prevalence, directly affecting animal production [3]. The high prevalence of fascioliasis in cattle was conveyed in all areas and is a severe problem in many nations [4-6]. The disease causes substantial financial losses to the livestock productiveness because of reduced output, liver condemnation, and reduced carcass value [7,8].

Above 17 million individuals are affected worldwide, where humans become accidental hosts by ingestion of contaminated aquatic vegetation or infrequently through ingesting of raw or undercooked liver products [9]. The topographical spreading of fascioliasis is intensely related to climate and ecological conditions such as the presence of water bodies, pastures, and wetlands. These conditions create an advantageous environment for the growth and spread of free-living fluke stages and for the growth of the intermediate host snail [10,11]. Separately from climate and ecological factors, animal level factors, for instance, age and breed and herd level factors such as stocking rate and type of farming system are also allied with the occurrence of the infection [12,13].

Several risk factors have been associated with fascioliasis in farm animals [14-16]. Nevertheless, evidence on the prevalence and risk factors associated with fascioliasis in cattle in Egypt is still limited.

Fascioliasis among animals and human in Egypt is of a great public health concern due to its clinical and epidemiological impacts. The Egyptian Academy of Scientific Research and Technology reported that the annual losses due to animal fascioliasis in Egypt were estimated at 190 million Egyptian pounds. All over the Egyptian governorates as well as newly reclaimed desert lands, both acute and chronic cases of animal fascioliasis have been reported [17,18]. Human infection causes serious hepatic pathological lesions in the liver cells due to migration of immature flukes. The disease affects the general health and immune status of the animal, and there is no accurate method for early diagnosis before the time of egg deposition adopted [19,20].

In Egypt, many factors enhanced the persistence of fascioliasis: The suitability of the climate and canals for the intermediate host; the resistance of metacercariae for dissociation, especially with the presence of shallow water, enough vegetation, and/or humidity; and continued exposure of the animals to encysted metacercariae, grazing habits, and movement between the infected and treated localities [20].

In 1988, the Egyptian Ministry of Agriculture reported that the mean percentage of *Fasciola* infestation (by fecal examinations) throughout the country reached 25.8%, and in the period from 1994 to 1997, the overall rates of fascioliasis among the slaughtered animals were 2.02% for sheep and goats, 3.54% for cattle, and 1.58% for buffaloes [21].

Therefore, this study focuses on the risk factors associated with the prevalence of fascioliasis affecting cattle population in three represented provinces of the Nile Delta of Egypt. In addition, the second aim is to estimate the economic losses as a result of fascioliasis.

## Materials and Methods

### Ethical approval

The study was approved by the Local Governmental Authority for Veterinary Services in Kafr El-Sheikh, El-Beheira, and Alexandria governorates and the Ethics and Welfare Committees of both faculties of Veterinary Medicine in Kafrelsheikh University and Damanhour University, Egypt, which is in accordance with the Institutional Animal Care.

### Area and design of the study

**Figure-1:**
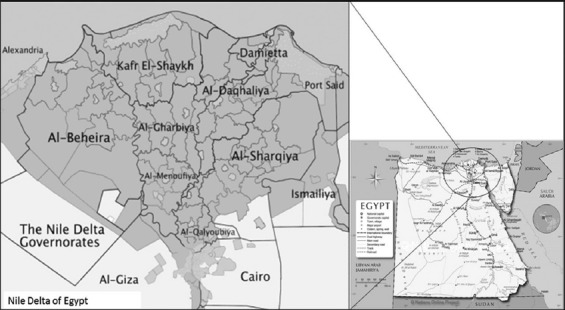
The map of Egypt (to the right) and the study area that includes three provinces: Kafr El-Shaykh, El-Beheira, and Alexandria (to the left) that contain a total of randomly selected 21 farms including 8 farms in Kafr El-Shaykh, 7 farms in El-Beheira, and 6 farms in Alexandria.

This study was conducted on three provinces (Kafr El-Shaykh, El-Beheira, and Alexandria; Figure-1). A total of randomly selected 21 farms including 8 farms in Kafr El-Shaykh, 7 farms in El-Beheira, and 6 farms in Alexandria were selected for data collection for this study. These provinces constitute a major proportion of cattle population in Egypt.

These selected farms were under supervision of visiting or resident veterinarians. The current study was conducted over a period extending from January 2015 to the end of December 2015.

Cattle were examined by the farm’s veterinarians regularly. Those animals exhibited signs such as poor body condition, rough coat, poor appetite, icteric mucous membrane of the eye, vulva with varying degree; decreased milk production, persistent diarrhea, and weight loss were suspected to be infected with liver flukes and were subjected to the parasitological examination.

### Sample collection and parasitological examination

A total of 4976 fresh fecal samples (≈400-450 sample/month) were collected from animal’s rectum individually in a dry clean polythene bags, labeled properly, and kept in 4°C. The collected fecal samples were processed in the laboratory by standard direct smear and sedimentation techniques as described previously [22,23] for the detection of *Fasciola* spp. eggs.

Fecal samples with even one *Fasciola* spp. egg were considered as positive for fascioliasis. The eggs of *Fasciola* spp. were identified microscopically according to the key described before [22,24]. Briefly, under the microscope, the *Fasciola* spp. eggs were operculated, thin-walled, and yellow-brown stained. The identified operculated *Fasciola* spp. eggs were differentiated from those of *Paramphistomum* spp. which is somewhat larger and do not stain yellow, have a transparent shell, a much more distinct operculum, and well-defined embryonic shell.

### Collection of epidemiological data

Epidemiological data reported in a pre-set questionnaire including data on animal ID, age, sex, breed, body weight, health status/body condition, and milk production were recorded as well the farmers’ age, level of education, and the type of the farm such as organic or conventional farm.

Age of the cattle was determined on the basis of farm’s records or by dentition [25,26]. Animals were categorized into three age groups as young (<2 years), 2-3 years, and adult (>3 years). Health status of cattle was measured by body condition score (BCS) by observing the condition of tail head and loin areas and was classified into three groups as good (3-4), medium (2-3), and poor (1-2) [27]. Those cattle got the prophylactic treatment previously were recognized having prophylactic treatment.

Friesian, Holstein, and Holstein-Friesian cattle breeds are known to be imported from the United Kingdom, USA, and Europe, respectively, and were classified and kept by the farmers’ records under these breed’s names.

The selected cattle farms were grouped into two groups: Organic farms and conventional, according to the method described by Sorge *et al*. [28].

Age and the education level of the owner farmers were recorded at the time of the attending veterinarian, and the education level was not changed during the period of the study.

Data of weather condition including the average temperature and relative humidity were monthly recorded from internet websites [29,30] for each study region.

### Economic effects of fascioliasis

For calculation of the economic impacts of fascioliasis on the farms, three parameters were considered: Reduction in body weight, reduction in milk production, and the treatment costs for *Fasciola* positive animals according to the method described by El-Tahawy [31].

Body weight of *Fasciola* positive cattle was compared to the *Fasciola* negative cattle based on of the market price (4.07 USD/kg live body weight).

To estimate the effect of fascioliasis on milk production, farm’s records of daily milk production (kg/day) of each of all lactating cows were documented 1 month before the date of sample collection. The animals and their milk production values were then analyzed on the basis of two groups such as positive and negative to fascioliasis. The following formula was applied to calculate the percent of reduction in milk production: Difference of milk production between *Fasciola* positive and negative cow’s×100/Average milk production of *Fasciola* negative cows.

The reduction in milk for the dairy cow was calculated according to the price of milk (0.43 USD/kg milk). Cost of the treatment of *Fasciola* depends on the cost of the fasciolicidal drug used.

### Statistical analysis

Odds ratios (OR) for infection with fascioliasis were assessed using logistic regression analysis. An univariate analysis was conducted to check if there is an association between the dependent variable (fascioliasis) and independent variables (potential risk factors) and nominated only those that affect the dependent variable significantly (p<0.05). All independent variables passed this first screening were considered for the multiple logistic regression models. Hosmer and Lemeshow goodness of fit test was used to decide whether the independent variable should be a linear or categorical variable and consequently sex of the animal, age, and education level of the farmer and the provinces were excluded due to poor fit with the final model. The significance level of the final model was set as 0.05. Economic data were analyzed using T-independent test between the healthy and diseased animals. All statistical analyses were performed using SPSS version 24. (IBM).

## Results

Within the examined cattle population, Table-1 displayed the descriptive statistics, and the prevalence of fascioliasis with an overall prevalence of fascioliasis in the Nile Delta region of Egypt was 9.77%. Table-1 also showed that the mostly affected breed was Holstein Friesian (13.43%) than the Holstein breed (6.36%), while the male cattle (bulls) were more infected (10.82%) than females (8.37%). Furthermore, the most affected age is that <2 years (10.91%). Kafr El-Shaykh was recording a higher prevalence of 11.67% than El-Beheira (10.6%) and Alexandria (7.15%). Furthermore, the conventional farm was a higher prevalence of 12.53% than an organic one. Application of prophylactic treatment recorded lower prevalence (8.99%) than those that did not get prophylactic treatment (12.13%). The higher prevalence of fascioliasis was recorded in the humidity range of 50-60% and also in higher temperatures >31°C (12.16% and 13.01%, respectively).

**Table-1:** Descriptive statistics of the cattle population and prevalence of *Fasciola* infection in relation to the potential risk factors.

Epidemiological factors/parameters	Number of cattle in the population (%)	Number *Fasciola*-positive cattle	Prevalence (%)
Total	4976 (100.0)	486	9.77
Breed			
Holstein*	1971 (39.61)	125	6.36
Holstein Friesian*	1756 (35.29)	236	13.43
Friesian*	1249 (25.10)	125	10.00
Sex			
Male	2828 (56.83)	306	10.82
Female	2148 (43.17)	180	8.37
Age			
2-3	1823 (36.64)	173	9.95
>3	1210 (24.32)	101	8.35
<2	1943 (39.04)	212	10.91
Provinces			
El-Beheira	1754 (35.25)	186	10.60
Kafr El-Shaykh	1543 31.01	180	11.67
Alexandria	1679 (33.74)	120	7.15
Type of farm			
Organic	1864 (37.46)	96	5.15
Conventional	3112 (62.54)	390	12.53
Body condition score			
Medium	1624 (32.64)	177	10.90
Poor	1500 (30.14)	230	15.33
Good	1852 (37.22)	79	4.26
Application of prophylactic treatment			
Applied	3739 (65.41)	336	8.99
Not applied	1237 (34.49)	150	12.13
Age of farmer			
40-50	1369 (27.51)	123	8.98
>50	1418 (28.50)	95	6.70
<40	2189 (43.99)	278	12.70
Education level of the farmer			
Higher (middle school up to university)	1155	175	15.15
Basic (elementary school or lower)	3821	311	8.14
Relative humidity			
50-60	1562 (31.39)	190	12.16
>60	1917 (38.53)	130	6.78
<50	1497 (30.08)	166	11.08
Temperature			
26-31	1565 (31.45)	175	11.18
>31	1452 (29.18)	189	13.01
<26	1959 (39.37)	122	6.22

* Friesian, Holstein, and Holstein-Friesian cattle are known to be imported from the United Kingdom, USA, and Europe, respectively, and were classified and kept by the farmers’ records under these breed’s names

The sedimentation technique records higher prevalence than direct smear in all provinces of the study. Direct smear recording 5.13%, 6.5%, and 2.9% while sedimentation technique recording 10.6%, 11.67%, and 7.15% in El-Beheira, Kafr El-Shaykh, and Alexandria, respectively (Table-2).

**Table-2:** Comparison of prevalence rates according to parasitological examination technique used.

Parasitological technique used						

Location	Direct smear			Sedimentation technique		
	
	Number of examined animals	Number of positive animals for *Fasciola* spp. eggs	(%)	Number of examined animals	Number of positive animals for *Fasciola* spp. eggs	(%)
El-Beheira	1754	90	5.13	1754	186	10.6
Kafr El-Shaykh	1543	100	6.5	1543	180	11.67
Alexandria	1679	50	2.9	1679	120	7.15
Total	4976	240	4.82	4976	486	9.77

Table-3 summarizes the association between bovine fascioliasis and the potential risk factors in the final model of logistic analysis after conducting the univariate analysis (Table-4) and excluding the poor fit variables from the final model.

**Table-3:** Multiple logistic regression (final model) analysis of the risk factors associated with the prevalence of bovine fascioliasis in the Nile Delta region of Egypt.

Independent variables	Categories	Coefficient	Odds ratio	95% CI	p value
		1.25	-		0.0001
Breed	Holstein*	−0.96	2.1	1.11-2.19	0.001
	Holstein Friesian*	0.42	2.63	1.69-1.88	0.0001
	Friesian* (reference)	0	1		
Age	2-3	−0.86	1.99	1.96-2.01	0.0001
	>3	−1.01	1.52	1.33-1.71	0.0001
	<2 (reference)	0	1		
Type of farm	Organic	1.23	1.83	1.55-2.11	0.0001
	Conventional (reference)	0	1		
Body condition score	Medium	0.55	0.64	0.53-0.75	0.0001
	Poor	1.03	1.86	1.23-2.14	0.0001
	Good (reference)	0	1		
Application of prophylactic treatment	Applied	−0.60	0.71	0.52-0.89	0.001
	Not applied (reference)	0	1		
Relative humidity	50-60	0.60	0.86	0.76-0.96	0.0001
	>60	0.77	1.79	1.40-2.18	0.0001
	<50 (reference)	0	1		
Temperature	26-31	1.20	2.30	1.80-2.80	0.0001
	>31	1.01	1.68	1.15-2.20	0.0001
	<26 (reference)	0	1		

* Friesian, Holstein, and Holstein-Friesian cattle are known to be imported from the United Kingdom, USA, and Europe, respectively, and were classified and kept by the farmers’ records under these breed’s names. CI=Confidence interval

**Table-4:** Results of univariable logistic regression analysis between the potential risk factors and the prevalence of bovine fascioliasis in the Nile Delta region of Egypt.

Independent variables	Categories	Coefficient	Odds ratio	95% CI	p value	Pseudo-R^2^	Goodness of fit
		0.98	-		0.01		
Breed	Holstein*	−0.38	0.54	0.49-0.58	0.01	0.082	1
	Holstein Friesian*	0.43	0.55	0.42-0.68	0.001		
	Friesian* (reference)	0	1				
Sex	Male	0.72	0.42	0.37-0.46	0.01	0.147	0
	Female (reference)	0	1				
Age	2-3	−0.10	0.47	0.40-0.54	0.01	0.181	1
	>3	−0.25	0.24	0.18-0.31	0.05		
	<2 (reference)	0	1				
Provinces	Beheira	0.01	0.77	0.45-1.10	0.47	0.001	0
	Kafr El-sheikh	0.12	0.71	0.25-1.18	0.39		
	Alexandria (reference)	0	1				
Type of farm	Organic	−0.41	0.45	0.36-0.54	0.01	0.051	1
	Conventional (reference)	0	1				
Body condition score	Medium	0.77	1.57	1.03-2.11	0.0001	0.004	1
	Poor	1.41	1.81	1.48-2.13	0.0001		
	Good (reference)	0	1				
Application of prophylactic treatment	Applied	−0.61	0.63	0.51-0.74	0.001	0.031	1
	Not applied (reference)	0	1				
Age of farmer	40-50	−1.4	0.24	0.18-0.40	0.0001	0.10	0
	>50	−1.78	0.16	0.09-0.28	0.0001		
	<40 (reference)	0	1				
Education level of the farmer	Basic (elementary school or lower)	1.20	2.52	1.72-3.33	0.001	0.171	0
	Higher (middle school up to university) (reference)	0	1				
Relative humidity	50-60	0.02	1.02	0.76-1.36	0.87	0.029	1
	>60	0.48	1.62	1.20-2.19	0.001		
	<50 (reference)	0	1				
Temperature	26-31	0.82	2.29	1.73-3.03	0.0001	0.011	1
	>31	0.94	2.56	1.83-3.59	0.0001		
	<26 (reference)	0	1				

* Friesian, Holstein, and Holstein-Friesian cattle are known to be imported from United Kingdom, USA, and Europe, respectively, and were classified and kept by the farmers’ records under these breed’s names. CI=Confidence interval

Fascioliasis was diverse between different breeds. Conversely, Holstein Friesian breeds have a higher risk of having fascioliasis than the Friesian breeds (OR: 2.63, 95% confidence interval [CI]: 1.69-1.88). The prevalence of fascioliasis is significantly lower in cattle older than 3 years (OR: 1.52, 95% CI: 1.33-1.71) in comparison to those younger than 2 years. With regard to the type of farm, cattle raised on organic farms were associated with lower risk of fascioliasis than those raised on conventional farms (OR: 1.83, 95% CI: 1.55-2.11). In terms of BCS, cattle with medium and poor conditions were associated with higher risk of fascioliasis than those with good body condition (OR: 0.64, 95% CI: 0.53-0.75 and OR: 1.86, 95% CI: 1.23-2.14; respectively). Cattle that got prophylactic treatment were associated with lower risk of fascioliasis than those that did not get prophylactic treatment (OR: 0.71, 95% CI: 0.52-0.89).

With reference to the effect of the ecological conditions on the fascioliasis, cattle raised in farms located in areas with average relative humidity ranged 50-60% were associated with higher risk of fascioliasis than those raised in farms located in area with a relative humidity <50% with no significant difference. On the other side, those cattle raised in farms located in areas with relative humidity over 60% were associated with higher risk of fascioliasis than those raised farms located in areas with a relative humidity <50% (OR: 1.79, 95% CI: 1.40-2.18). Cattle raised in located in areas with temperature ranged 26-31°C and over 31°C were associated with higher risk of fascioliasis than those raised in areas with temperature <26°C (OR: 2.30, 95% CI: 1.80-2.80 and OR: 1.68, 95% CI: 1.15-2.20; respectively).

In regard to the economic losses as a result of *Fasciola*, data in Table-5 revealed that *Fasciola*-positive animal showed lower in their live body weights than those *Fasciola*-free (317.35 kg/animal vs. 366.22 kg/animal, respectively, p<0.05). Conversely, *Fasciola*-free animals have more prominent normal milk yield than those practically identical infected ones (27.10 kg/day vs. 32.01 kg/day, p<0.05). The total monetary losses as a result of fascioliasis were estimated as the loss in body weight totaled with the estimated loss in milk production and the cost of treatment. The normal estimation of the weight reduction evaluated for single cattle was 211.3 USD, where the cost of 1 kg was 4.07 USD. Likewise, the mean estimation of the loss in milk production per individual *Fasciola*-positive cattle was assessed 210.5 USD, where the cost of 1 kg of milk was 0.43 USD. Totaling to the treatment cost equal to 8.5 USD/cow, the total monetary losses per individual *Fasciola*-positive cattle were evaluated 221.2 USD/cow.

**Table-5:** Economic effects of bovine fascioliasis in the Nile Delta region of Egypt.

Variable	*Fasciola*-positive cattle		*Fasciola*-negative cattle	
	
	Number	M±SEM	Number	M±SEM
Total weight (kg)	486	317.35±1.13*	4490	366.22±1.07*
Milk production (kg)	180	27.00±0.11*	1986	32.01±0.09*
Weight reduction value (USD)	486	210.54±0.14	4490	-
Milk reduction value (USD)	180	2.13±0.05	1986	-
Treatment cost (USD)	486	8.53±0.08	4490	-
Total monetary losses (USD)	486	221.21±0.17	4490	-

* Means are significantly different at p<0.05. M=Mean; SEM=Standard error of mean

## Discussion

In Egypt, *F. hepatica* and *F. gigantica*, the causes of fascioliasis, are prevalent among livestock in the Nile Delta [6,32] and greatly affect the livestock production in the developing countries, especially in Egypt. Biu *et al*. [33] reported that fascioliasis causes great losses in the form of poor feed conversion, weight loss, slow fattening, and reduced milk yield, reproductive failure, and ultimately death.

Focusing on the potential risk factors associated with the spread of fascioliasis among the livestock animals may help on understanding the transmission and also may be benefit for the control strategy of fascioliasis.

The overall prevalence of fascioliasis in cattle was 9.77%, and this obtained estimate was lower than those recorded by the studies of Haridy *et al*. [21] and ElKhtam and Khalafalla [6], who reported that the prevalence of fascioliasis in farm animals was 5.3% and 5.8%, respectively. While Bazh *et al*. [34] reported the prevalence among the infected cattle in El- Beheira reaches about 50%. The difference of *Fasciola* prevalence between the three provinces could be explained by the alterations of the climatic condition and the type of management and production systems followed inside each farm located in each province.

The risk of fascioliasis in cattle was diverse among different breeds. However, earlier research findings [35-37] found that the risk infections for all cattle breeds that graze on cultivated and natural pastures were equal within the same area of study as it was associated with the same management conditions.

On the contrary, in Bangladesh, higher prevalence of fascioliasis was reported in Hariana breed [38]. Similarly, Kato *et al*. [39] found a higher prevalence of fascioliasis in Japanese native cattle breed than Friesian or Jersey breeds because of the management applied for the cattle breeds. Friesians have partial contact to cultivated pastures, while Japanese native cattle breed graze both in natural pastures and cultivated rice field, which showed that rice straw feeding is proposed to be correlated with high rates of cattle fascioliasis in Japanese native cattle.

In regard to the effect of sex, the higher prevalence of fascioliasis in females than in males could be explained to the statement that most female cattle are kept for milking which considered as stressful physiological factor lowering the immunity against infections. These outcomes are in agreement with the studies conducted by Yildirim *et al*. [36], who investigated that the prevalence of hepatic fascioliasis was noted higher in females than males one. However, other studies mentioned that there was no statistical significant difference between the prevalence recorded in females comparing to that calculated in males [37,38,40] and they referred to the similarity on the management system between both sexes. Furthermore, Umbreen and Azhar [41] found a significant higher prevalence of fascioliasis in males than females and the explanation behind that is might be owing to keeping females under healthier management and nourishing conditions comparable with males which are kept free to touch the infections inside the fields.

Undoubtedly, the animals which did not receive prophylactic treatment for fascioliasis are of higher risk of fascioliasis than those treated. These outcomes supported by the findings of Kheider [42] who specified that the higher predominance of fascioliasis was observed in cattle did not get the prophylactic treatment.

Based on body condition, fascioliasis infection rate among cattle was statistically analyzed to investigate the impact of the disease on emaciated infected animals. The significantly lower prevalence of fascioliasis connected with lower odds was distinguished on those of good BCS than medium and poor BCS. The current results agreed with those of Bekele *et al*. [43] and Kheider [42].

Concerning the type of the farms located in the three provinces, it has been investigated that the conventional farms are of higher risk of fascioliasis than organic ones which could be referred to that the traditional farms did not follow control measures for fascioliasis as organic ones. These findings and explanation are consistent with the report described by Yildirim *et al*. [36].

With respect to the educational level and the age of the owner, it has been noticed that the prevalence of fascioliasis for cattle raised by basic level is higher than those raised by higher one, and this distinction is significant. This might be cleared up as higher educated farmers are well-informed, modernized, and much organized in raising and noticing farm animals than lower educated farmers.

Environmental conditions have a part in the spreading of fascioliasis. Our obtained results are coordinated with the result by Al-Jibouri *et al*. [44], who found that the prevalence of fascioliasis in cattle was associated with lower temperature and higher relative humidity. Urquhart *et al*. [45] specified that moisture, optimal temperature, and suitable snail habitat are main factors inducing the occurrence of fascioliasis in a certain area. Furthermore, the optimal temperatures of 10°C and 16°C are necessary for infection of snail vectors with *Fasciola* spp. and for the next developments to produce cercariae.

In this study, the economic losses that associated with the fascioliasis were assessed in terms of loss in milk production, reduction in body weight, and cost of treatment. Our estimated losses are supported by investigations of Gavinho *et al*. [46] who confirmed that fascioliasis could represent a notable (p=0.004) diminishing of 5.8% of body weight among contaminated and non-contaminated cows, resulting in 35.00 USD loss/head of income. It was also determined that animals can be reduced between 8 and 28% of its body weight when experimentally infected with *Fasciola* spp., compared with the control group [47]. Furthermore, Elmonir *et al*. [48] expressed that the total economic losses because of fascioliasis as far as liver condemnations and carcasses weight losses over the 3 years of the study were evaluated 16,800.4 USD. In addition, Degheidy and Al-Malki [49] reported a yearly loss of 20,000 USD because of animal fascioliasis at Taif region, Kingdom of Saudi Arabia.

## Conclusion

In this study, the epidemiological investigation of risk factors confirmed that there was a significant association between the prevalence of fascioliasis with the farm location, sex, breed, age of the animal, BCS, type of farm, frequency of anthelmintic treatment, environmental conditions, and farmer status. In addition, the fascioliasis caused great losses in cattle farms through reduction in both body weight and milk production. As well, these outcomes could be helpful for planning of strategies to control fascioliasis in Egypt.

## Authors’ Contributions

All authors contributed in the planning and doing research work as follows: ASE (study design, doing, statistics, and writing), EKB (study design, doing, and writing), and REK (study design, doing, writing, and publishing). All authors read and approved the final manuscript.

## References

[1] Cawthorne R.J.C (1984). Anthelminthics for Cattle, Sheep, Goats, Pigs, Horses and Poultry.

[2] Soliman M.F (2008). Epidemiological review of human and animal fascioliasis in Egypt. J. Infect. Dev. Ctries.

[3] Olsen A, Frankena K, Bødker R, Toft N, Thamsborg S.M, Enemark H.L, Halasa T (2015). Prevalence, risk factors and spatial analysis of liver fluke infections in Danish cattle herds. Parasit. Vectors.

[4] Torgerson P, Claxton J, Dalton J.P (1999). Epidemiology and control. Fasciolosis.

[5] Tum S, Puotinen M.L, Skerratt L.F, Chan B, Sothoeun S (2007). Validation of a geographic information system model for mapping the risk of fasciolosis in cattle and buffaloes in Cambodia. Vet. Parasitol.

[6] Elkhtam A.O, Khalafalla R.E (2016). Surveillance of helminthes and molecular phylogeny of *Fasciola gigantica* infecting goats in Sadat district, Egypt. Int. J. Sci. Res. Sci. Technol.

[7] Kaplan R.M (2001). *Fasciola hepatica*:A review of the economic impact in cattle and considerations for control. Vet. Ther.

[8] Vazquez M.J.S, Lewis F.I (2013). Investigating the impact of fasciolosis on cattle carcass performance. Vet. Parasitol.

[9] Hopkins D.R (1992). Homing in on helminths. Am. J. Trop. Med. Hyg.

[10] Charlier J, Bennema S.C, Caron Y, Counotte M, Ducheyne E, Hendrickx G (2011). Towards assessing fine-scale indicators for the spatial transmission risk of *Fasciola hepatica* in cattle. Geospat. Health.

[11] Relf V, Good B, Hanrahan J.P, McCarthy E, Forbes A.B, Dewaal T (2011). Temporal studies on *Fasciola hepatica* in *Galba truncatula* in the west of Ireland. Vet. Parasitol.

[12] Kuerpick B, Conraths F.J, Staubach C, Frohlich A, Schnieder T, Strube C (2013). Seroprevalence and GIS-supported risk factor analysis of *Fasciola hepatica* infections in dairy herds in Germany. Parasitology.

[13] Petros A, Kebede A, Wolde A (2013). Prevalence and economic significance of bovine fasciolosis in Nekemte municipal abattoir. J. Vet. Med. Anim. Health.

[14] Theodoropoulos G, Peristeropoulou P, Kouam M.K, Kantzoura V, Theodoropoulou H (2010). Survey of gastrointestinal parasitic infections of beef cattle in regions under mediterranean weather in Greece. Parasitol. Int.

[15] Kantzoura V, Diakou A, Kouam M.K, Feidas H, Theodoropoulou H, Theodoropoulos G (2013). Seroprevalence and risk factors associated with zoonotic parasitic infections in small ruminants in the Greek temperate environment. Parasitol. Int.

[16] Kantzoura V, Kouam M.K, Theodoropoulou H, Feidas H, Theodoropoulos G (2012). Prevalence and risk factors of gastrointestinal parasitic infections in small ruminants in the Greek temperate mediterranean environment. Open J. Vet. Med.

[17] Lotfy W.M, Hillyer G.V (2003). *Fasciola* species in Egypt. Exp. Pathol. Parasitol.

[18] El-Shazly A.M, El-Nahas H.A, Soliman M, Sultan wD.M, Abedl-Tawab A.H, Morsy T.A (2006). The reflection of control programs of parasitic diseases upon gastrointestinal helminthiasis in Dakahlia governorate, Egypt. J. Egypt. Soc. Parasitol.

[19] Haseeb A.N, El-Shazly A.M, Arafa M.A, Morsy A.T (2002). A review on fascioliasis in Egypt. J. Egypt. Soc. Parasitol.

[20] El-Bahy N.M (1998). Strategic control of fascioliasis in Egypt. Review Article:Submitted to the Continual Scientific Committee of Pathology, Microbiology and Parasitology.

[21] Haridy F.M, Ibrahim B.B, Morsy T.A, El-Sharkawy I.M (1999). Fascioliasis an increasing zoonotic disease in Egypt. J. Egypt. Soc. Parasitol.

[22] Soulsby E.J.L (1983). Helminths, Arthropods and Protozoa of Domesticated Animals.

[23] Hanson J, Perry B (1994). The Epidemiology, Diagnosis and Control of Helminth Parasites of Ruminants:A Hand Book.

[24] Thienpont D, Rochette F, Vanparijs O.F.J (1986). Diagnosing Helminthiasis by Coprological Examination.

[25] Delahunt A, Habel R.E (1986). Applied Veterinary Anatomy.

[26] Pace J.E, Wakeman D.L (2003). Determining the Age of Cattle by Their Teeth.

[27] Nicholson M.J, Butterworth M.H (1986). A Guide to Condition Scoring of Zebu Cattle.

[28] Sorge U.S, Moon R, Wolff L.J, Michels L, Schroth S, Kelton D.F, Heins B (2016). Management practices on organic and conventional dairy herds in Minnesota. J. Dairy Sci.

[29] Underground W (2015). Weather Underground.

[30] Meteo F (2015). Hourly Weather History for Egypt.

[31] El-Tahawy A.S (2010). The prevalence of selected diseases and syndromes affecting Barki sheep with special emphasis on their economic impact. Small Rumin. Res.

[32] Britain G, MAF Ministry of Agriculture and Fisheries (1986). Manual of Veterinary Parasitological Laboratory Techniques.

[33] Biu A, Paul B, Konto M, Ya'Uba A (2013). Cross sectional and phenotypic studies on fasciolosis in slaughter cattle in Maiduguri. J. Agric. Vet. Sci.

[34] Bazh E.K, Beder N.A, Ayoub M, Sadek K (2012). *Fasciola* Infection Among Cattle and Buffaloes at Behera Governorate. Egypt. Zagazig Vet. J.

[35] Sanchez-Andrade R, Paz-Silvaa A, Suarez J.L, Panadero R, Pedreira J, Lopez C, Diez-Banos P, Morrondo P (2002). Influence of age and breed on natural bovine fasciolosis in an Endemic area (Galicia, NW Spain). Vet. Res. Commun.

[36] Yildirim A, Duzlu O, Inci A (2007). Prevalence and risk factors associated with *Fasciola hepatica* in cattle from Kayseri province, Turkey. Rev. Méd. Vét.

[37] Kanyari P.W, Kagira J.M, Mhoma R.J (2010). Prevalence of endoparasites in cattle with zoonotic potential within urban and peri-urban areas of Lake Victoria basin, Kenya. Am. J. Agric. Biol. Sci.

[38] Kabir M.H, Eliyas M, Hashem M.A, Miazi O.F (2010). Prevalence of zoonotic parasitic diseases of domestic animals in different abattoir of Comilla and Brahman Baria region in Bangladesh. J. Zool.

[39] Kato M, Murakami Y, Shimizu M, Yamamoto H, Yonemoto Y, Ishii K, Kira S (2005). Survey of cattle Fascioliasis in tsuyama abattoir. Environ. Health Prev. Med.

[40] Khan M.K, Sajid M.S, Iqbal Z, Iqbal M.U (2009). Bovine fasciolosis prevalence, effects of treatment on productivity and cost benefit analysis in five districts of Punjab, Pakistan. Res. Vet. Sci.

[41] Umbreen J.K, Azhar M (2012). Prevalence of fasciolosis in cattle under different managemental conditions in Punjab. Pak. J. Zool.

[42] Kheider Z.A (2014). Prevalence and Risk Factors of Bovine Fasciolosis in North Kordofan State, Sudan. M.Sc, College of Veterinary Medicine, University of Khartoum.

[43] Bekele M, Haftom T, Yehenew G (2010). Bovine fasciolosis:Prevalence and its economic loss due to liver condemnation at Adwa municipal abattoir, North Ethiopia. Ethiop. J. Agric. Sci. Technol.

[44] Al-Jibouri S, Moayad M, Al-Mayah H, Hadi R.H (2011). The factors affecting metacercarial production of *Fasciola gigantica* from *Lymnaea auricularia* Snails. J. Basrah Res. Sci.

[45] Urquhart G.M, Armour J, Duncan J.L, Dunn A.M, Jennings F.W (1996). Veterniary Parasitology.

[46] Gavinho B, Kulek A.C.G, Molento M.B (2008). Quantitative determination and geographic distribution of *Fasciola hepatica* in bovines slaughtered in São Josédos Pinhais PR. XVI Encontro de Iniciação ar203˜aoCientificadaUFPR:Curitibað(CD-Rom).

[47] Malone J.B, Craig T.M (1990). Cattle liver flukes:Risk assessment and control. Compend. Contin. Educ. Pract. Vet.

[48] Elmonir W, Mousa W, Sultan K (2015). The prevalence of some parasitic zoonoses in different slaughtered animal species at abattoir in the mid-delta of Egypt;with special reference to its economic implications. Alex. J. Vet. Sci.

[49] Degheidy N, Al-Malki J (2012). Epidemiological studies of fasciolosis in human and animals at Taif, Saudi Arabia. World Appl. Sci. J.

